# Nodular Fasciitis – Fine Needle Aspiration Cytology Diagnosis and Its Pitfalls, with Review of Literature

**DOI:** 10.30699/IJP.14.1.76

**Published:** 2018-12-27

**Authors:** Padmapriya Jaiprakash, Balaji Radhakrishnan, Ranjini Kudva, Manna Valiathan, Seetharam Prasad

**Affiliations:** 1 *Associate Professor, Dept. of Pathology, Kasturba Medical College, Manipal Academy of Higher Education, Mani- pal, India*; 2 *Ex-Post-Graduate, Dept. of Pathology, Kasturba Medical College, Manipal Academy of Higher Education, Manipal, India*; 3 *Professor, Dept. of Pathology, Kasturba Medical College, Manipal Academy of Higher Education, Manipal, India*; 4 *Professor, Dept. of General Surgery, Kasturba Medical College, Manipal Academy of Higher Education, Manipal, India*

**Keywords:** Fasciitis, Fine-Needle Aspiration, Cytological Technic

## Abstract

**Background and Objective:**

Nodular fasciitis (NF) is a self-limiting, transient neo- plasm composed of fibroblasts and myofibroblasts. Since it regresses spontaneously, diagnosis by fine needle aspiration (FNA) cytology plays a major role in its management.

**Methods:**

We present a series of 8 cases with either FNA or biopsy diagnosis ofNF, and study the major cytological features with a review of literature on diagnostic criteria and its pitfalls.

**Results and Conclusion:**

The 8 cases occurred in patients between the age of 14 to 72 years, with equal sex distribution. FNA diagnosis concurred in 4 cases. Causes of wrong diagnosis included lack of clinical information and paucicellular smear. FNA cytology is an important tool in the diagnosis of nodular fasciitis, in appropriate clinico-radiologicalsetting.

## Introduction

Nodular fasciitis (NF) is a self-limiting neoplasm arising from fibroblastic or myofibroblastic cells. It usually oc- curs in the subcutaneous tissue and muscle occasionally. Most common sites include upper extremity, trunk, head, and neck. It affects both sexes equally, occurring more in young adults. 2 rare variants include intravascular fasciitis and cranial fasciitis (1, 2).

**Objectives: **To determine if fine needle aspiration cytology (FNAC) can be used with high specificity in the diagnosis of nodular fasciitis (NF), correlating with clinical presentations.

## Materials and Methods

Laboratory information system was used to retrieve all cases with a differential diagnosis of nodular fasciitis on FNAC or a definite diagnosis of the same on histopathology. The FNA slides were then reviewed to note the salient features on cytology. The results were analyzed and conclusions drawn.

## Results and Discussion

There were a total of 15 cases of NF, diagnosed during a period of 5 years from April 2012 to March 2017. The age of the patients ranged from 14 to 85 years. Incidence in males (8 in number) was slightly more than that in females (7). FNA was performed only in 8 cases. 5 cases had both FNA and biopsy while 3 had only FNA (case 1, 5 and 7). Of these, 6 cases had a differential of NF while one was diagnosed as just spindle cell lesion. One case suspected to be intraparotid lymph node was misdiagnosed as pleomorphic adenoma.(table 1)

**Table 1:** The results have been tabulated

Serial number	Age in years	Sex	Site	FNA diagnosis	Histopathological diagnosis
1	72	F	Soft tissue over Sternoclavicular joint	? Nodular fasciitis (NF)/ Schwannoma	-
2	24	M	Chest wall	NF/ Neurofibroma	NF
3	14	M	Intraparotid lymph node	Pleomorphic adenoma	NF
4	39	F	Neck lymph node	Spindle cell lesion	NF with extensive collagenisation
5	20	F	Leg	? NF/ proliferative fasciitis	Skin biopsy with descriptive report
6	31	M	Abdominal wall – epigastrium	Spindle cell lesion? NF	NF
7	27	M	Right arm	F	-
8	20	F	Groin	NF/ Neurofibroma	Benign fibroblastic/ myofibroblastic tumour (tru-cut)

**Cytological Findings**

All the FNA smears were cellular with tissue fragments with fibroblast-like spindle cells, myofibroblasts and ganglion cell-like cells (GCLCs), enmeshed in abundant myxoid background. The cells were elongated with scant cytoplasm, spindle nucleus with tapering ends, few with wavy configuration. Occasional bi-nucleate cells were seen. Identification of both tissue fragments and ganglion cell-like cells were seen with high specificity for the diagnosis.

The other frequently associated features included few inflammatory cells in the background, occasional mito- ses and isolated or single cells. One case showed multinucleated giant cells. These features may be used to make a definitive diagnosis of NF on FNA.

**Cases**

Case 1 was an old lady with swelling over the sternocleidomastoid joint who was subjected to FNA as first line of investigation. However, after the diagnosis as spindle cell lesion with a differential of NF or schwannoma, computed tomography was done which showed the involvement of clavicle, and the nature of the lesion labelled as malignant. However, on review also, FNA slides showed no features of malignancy. The patient was lost on follow-up.

Case 2 was a 24 year old male with chest wall swelling since a month, which was non-tender. Ultrasonography (USG) showed a solid lesion in subcutaneous plane with a probable neural origin. The FNA and histopathology diagnosis concurred in this case.

Case 3 was a 14 year old male with an intraparotid swelling which was suspected to be a lymph node on USG. FNA smears were paucicellular with few spindle cell clusters and occasional plasmacytoid/GCLCs. It was mis- diagnosed as pleomorphic adenoma. Excision biopsy showed features of NF.

Case 4 was a 39-year-old lady with suspected neck lymph nodal swelling. FNA was diagnosed as spindle cell lesion, which on review showed classical features of NF. Biopsy showed NF with extensive collagenization.

Case 5 was a 20 year old student with a subcutaneous nodule in leg which was suspected to be erythema no- dosum. A punch biopsy was done which was superficial and did not show any specific features. This was fol- lowed by FNA, which showed features of NF. Hence the patient was followed up on and the lesion regressed in 2 months.

Case 6 was a 31-year-old male with epigastric swelling with clinical diagnosis of fibroma. FNA showed cel- lular tissue fragments with classical features. However, due to the presence of occasional mitosis, a biopsy was requested which showed features of NF with osteoclastic giant cells.

Case 7 was a 27-year-old male with left arm swelling. FNA smears showed plump fibroblasts in a mixed in- flammatory background. A differential of inflammatory lesion/ NF was given. No biopsy was done. The lesion had regressed on a 3-month follow-up.

Case 8 was again a 20-year-old male student with a rapidly increasing swelling in groin. USG showed a well- defined heterogeneous hyperechoic lesion, suggestive of nodal mass. FNA showed cellular smears with tissue fragments composed of spindle cells and few GCLCs. However, since clinically it was rapidly increasing, a Tru-cut biopsy was performed which showed spindle cell lesion. Immunohistochemistry (IHC) showed tram track positivity for smooth muscle actin (SMA) and showed a negative result for S100 protein, confirming the myofibroblastic nature of the lesion. The patient was followed upon, withoutperforming surgical excision and was asymptomatic after 6 months.

**Figure 1:**
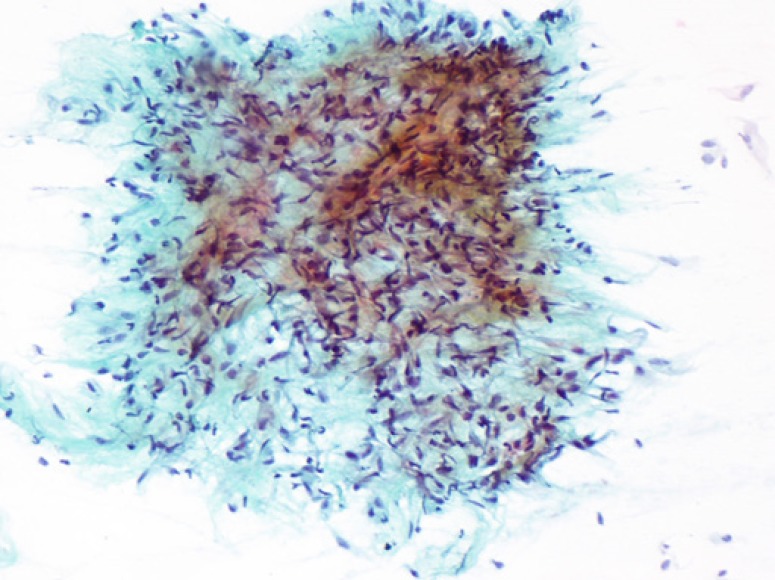
Cellular smears showing tissue fragments composed of spindle cells, fibroblasts and single cells in a myxoid back- ground. Papanicolaou stain, x100

**Figure 2:**
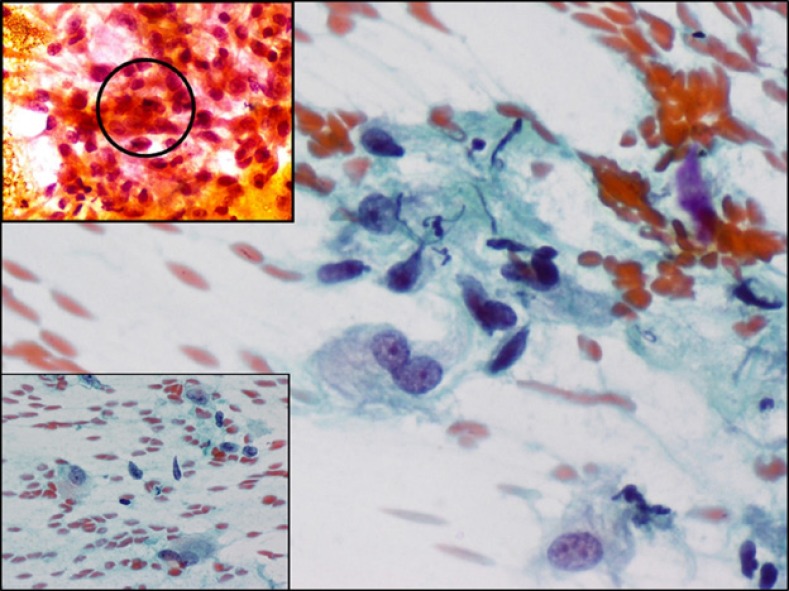
Large ganglion cell-like cells, including a binucleate one. Inset shows a mitoses (top) and histiocyte like cells (be- low). Papanicolaou stain, x200

**Figure 3:**
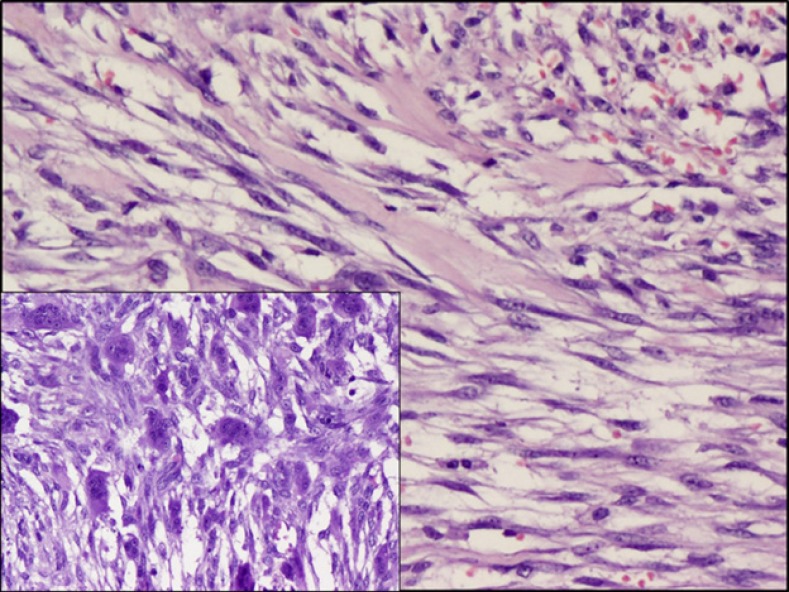
Fascicles of spindle cells hyalinised background (left); with interspersed osteoclastic giant cells (right). H&E, x200

**Figure 4:**
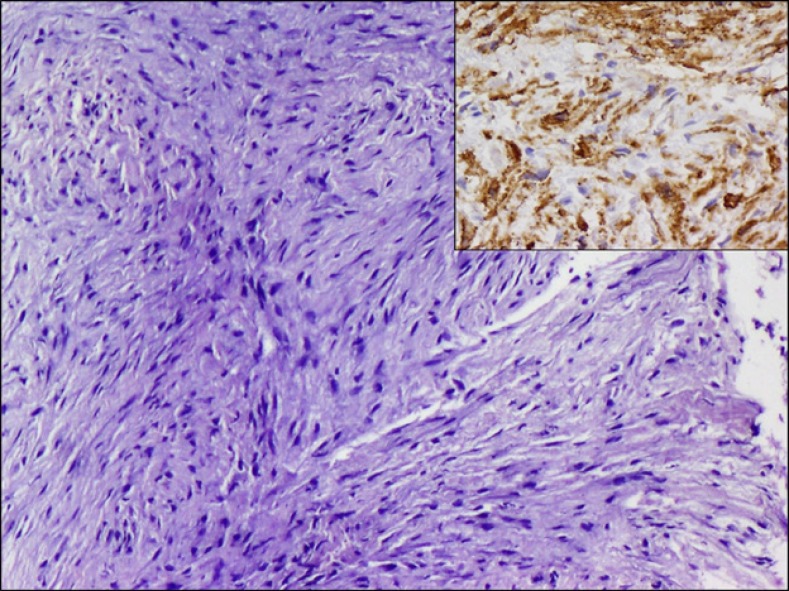
Section showing fascicles of spindle cells in a myxoid background, with SMA positivity on immunohistochemistry (Inset). H&E, x200, Inset DAB, x100

Nodular fasciitis was first described by Konwaler in 1955 (1). These lesions rapidly grow clinically, with a size varying from 2 to 5cms. It usually occurs in adolescents and young adults, in superficial tissues, easily accessible for FNA. Accurate diagnosis by FNA plays an important role in its management as it can just be followed up on by looking for regression and can be operated on only when clinical behavior is ominous.

Most cases of NF have typical cytological features. Smears are highly cellular with tissue fragment-like along with numerous isolated cells and GCLCs. Wong and Di have set the criteria for the identification of GCLC as being twice the size of plump fibroblasts. GCLCs are found to be specific for NF, proliferative fasciitis and proliferative myositis (3).

Smears are usually hypercellular, with tissue fragments composed of spindle cells with scant cytoplasm, elon- gated, oval, uniform nuclei, and granular chromatin in a myxoid background (1). There may be nuclear over- lapping of the cells focally. Though occasional cells may show anisonucleosis, pleomorphism is not a feature of NF. However, mitoses can be brisk, raising a false alarm during initial screening of the smears. Larger myo- fibroblastic cells though may appear stellate shaped or even multinucleated; they share the nuclear features of spindle cells (2).

One of the most important differential diagnoses is sarcoma. The diagnostic accuracy increases with no false positive result when correlated with clinical findings (3). NF along with related reactive myofibroblastic prolifera- tions regress spontaneously, and hence surgical intervention is not required. The other differentials include granu- lomatous diseases, fibrous histiocytoma, schwannoma, neurofibroma and fibromatosis. Mitoses can be frequent in NF. However, sarcomas show cells with marked anaplasia and atypical mitoses. Stellate to spindle cells in a myxoid background and GLCL should point towards reactive proliferation, and clinical details should be actively sought.

Granulomas may be differentiated by the slipper shaped nuclei of epithelioid cells and the absence of myxoid stroma characteristic of NF. Fibrous histiocytoma show spindle or rounded cells, along with hyalinised col- lagen bundles, chronic inflammatory cells, macrophages and touton giant cells (5). Schwannoma shows both hypocellular and cellular fragments with typical wavy nuclear configuration. Neurofibromas, on the other hand, are usually hypocelluar with loosely cohesive wavy spindle cells in a myxoid matrix (4). Fibromatosis yields cell poor aspirate with isolated spindle cells lacking nuclear pleomorphism with hyalinised collagenous stromal fragments (4).

In the head and neck region, it can be confused with salivary gland tumor occurring in specific sites due to the presence of spindle to plasmacytoid cells in a myxoid background, mimicking pleomorphic adenoma (PA) (6,7). A few points in favor of diagnosis of NF over pleomorphic adenoma include predominantly spindle cells with fuzzy cytoplasmic borders and multiple processes, prominent nucleoli, occasional mitoses, in contrast to the plas- macytoid cells of PA (7).

When available, cell block can be made from the aspirates and immunohistochemistry performed on them to confirm myofibroblastic nature (2).

Controversy always existed if NF is a reactive process or neoplastic. However, molecular and cytogenetic studies have shown numerous genetic aberrations, proving it to be a neoplastic process beyond doubt (8,9). Meng et al. went a step further and compared NF with myofibroblastic sarcoma (MS). They found a significantly lower number of genetic aberrations in NF (0.4) as comparted to MS (5.4), indicating a distinct genetic pathway in their development. Ericson-Johnson et al (9) have proved USP6 rearrangements with the formation of the fusion gene *MYH9-USP6 *occur in most examples of NF. They have describe NF as a self-limiting ‘transient neoplasia’ (10).

## Conclusion

FNA can be a valuable diagnostic method to help with the diagnosis of NF before excision, if it is interpreted together with clinical and radiologic findings.

Acknowledgement: We would like to acknowledge the technical staff of Dept. of Pathology, KMC, Manipal, especially Mr. Sathish Poojary and Mr. Prasanna Rao, for their constant support.

## Conflict of Interest

The authors declare that there is no conflict of interest in the publication of this paper..
